# Supplementary figures and images for: Metabolomic and Transcriptomic Analyses Reveal the Characteristics of Tea Flavonoids and Caffeine Accumulation and Regulation between Chinese Varieties (Camellia sinensis var. sinensis) and Assam Varieties (C. sinensis var. assamica)
Source: Genes (Basel). 2022 Oct 31;13(11):1994. doi: 10.3390/genes13111994 (PMC9690216; doi:10.3390/genes13111994)

Scores (OPLS-DA)

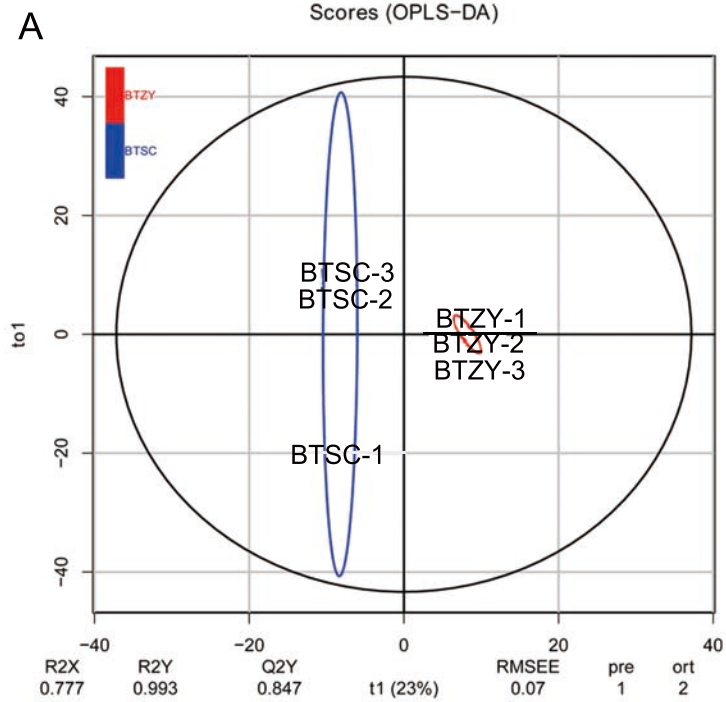

Scores (OPLS-DA)

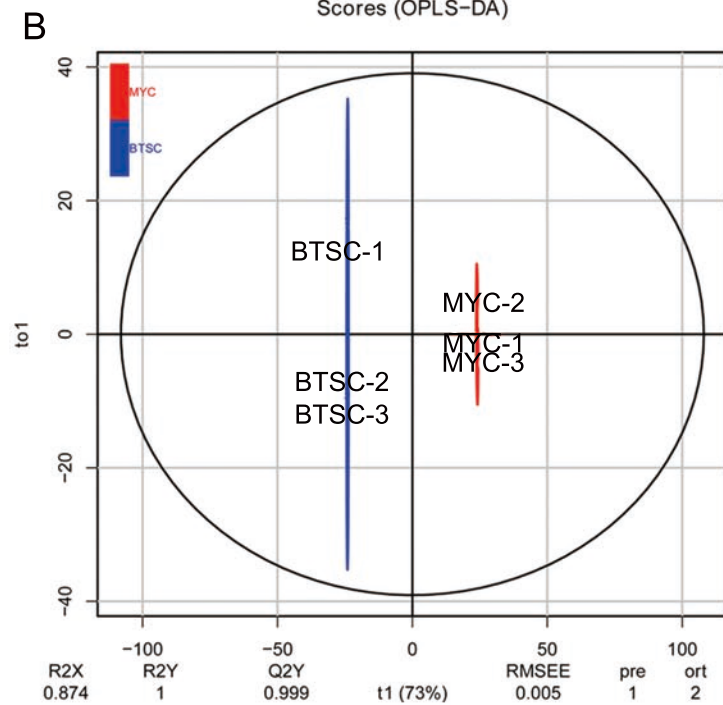

Scores (OPLS-DA)

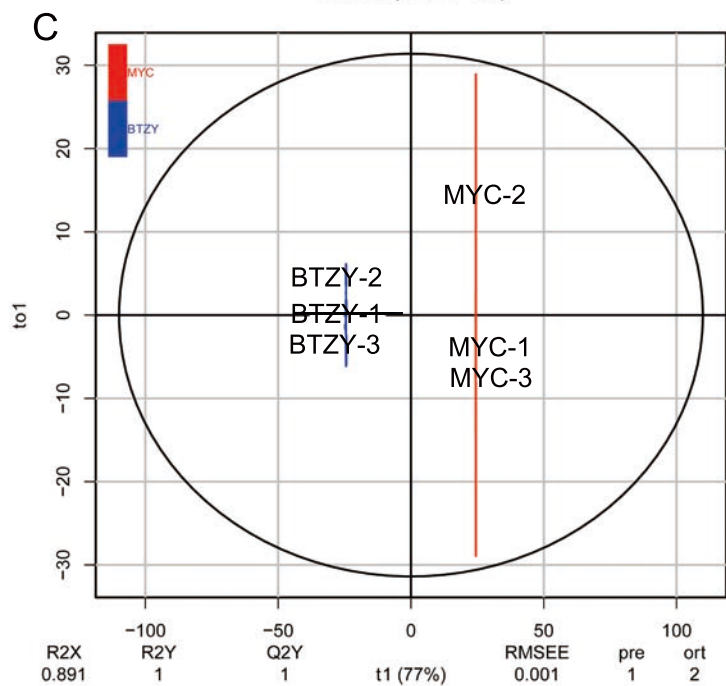

Supplement: Supplementary file 1 [file genes-13-01994-s001.zip › Figure S1.pdf]

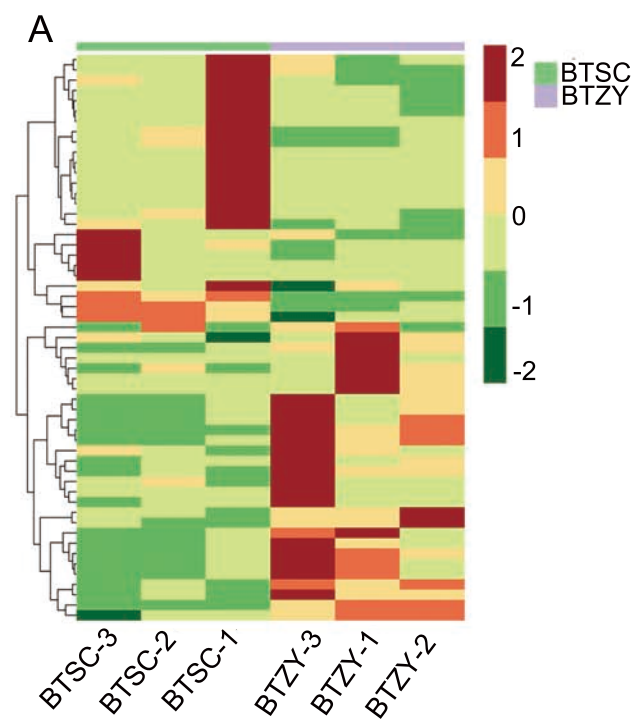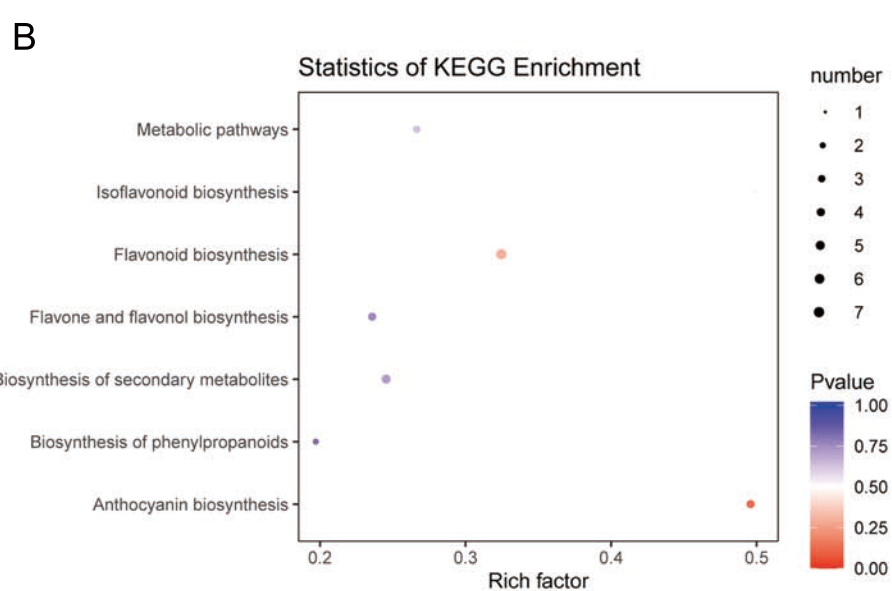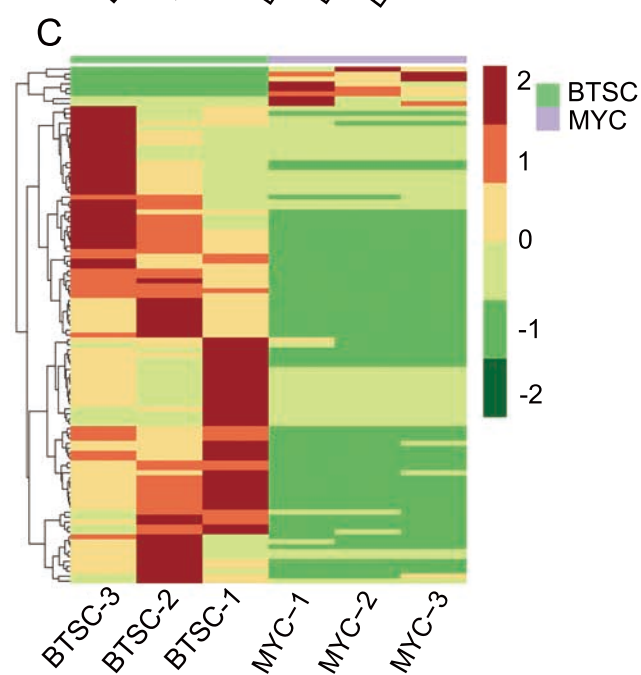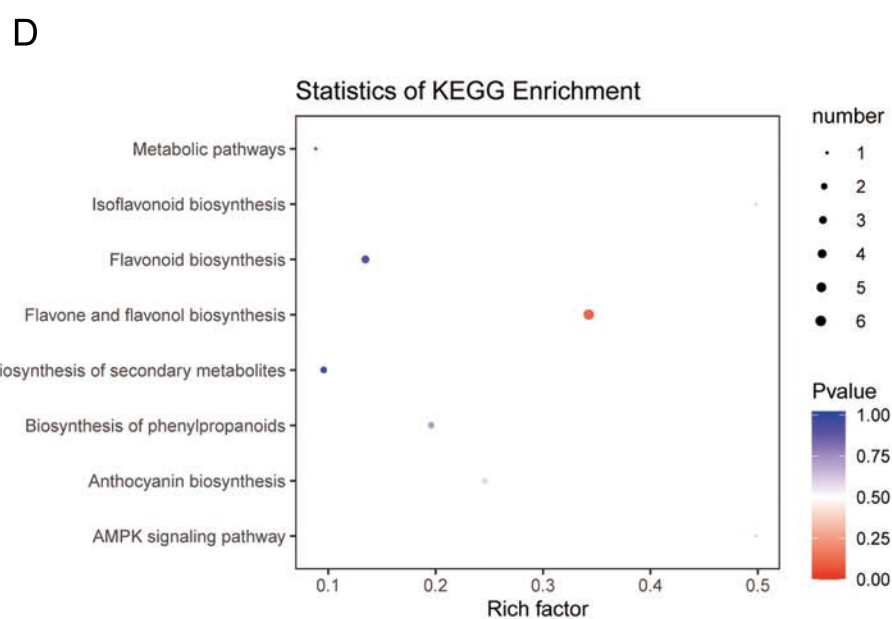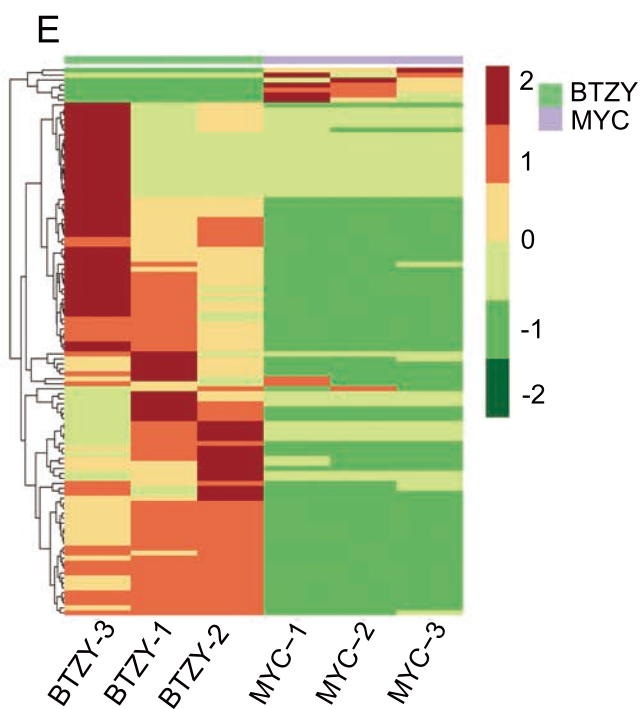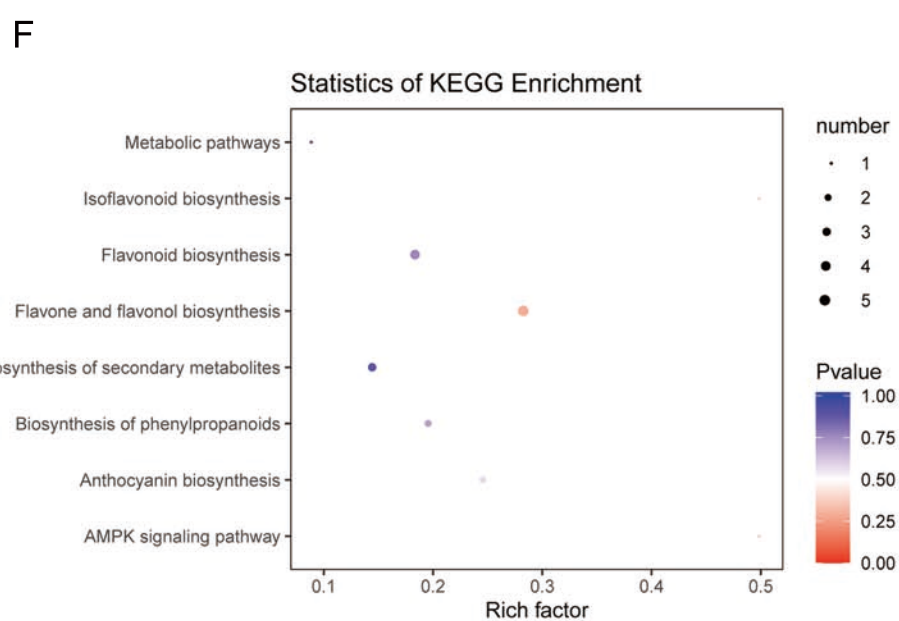

Supplement: Supplementary file 1 [file genes-13-01994-s001.zip › Figure S2.pdf]

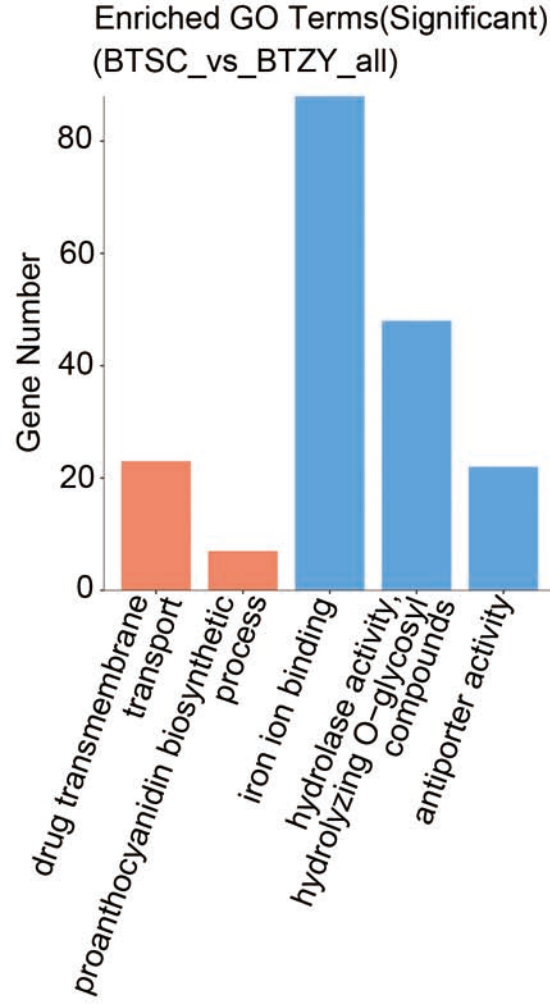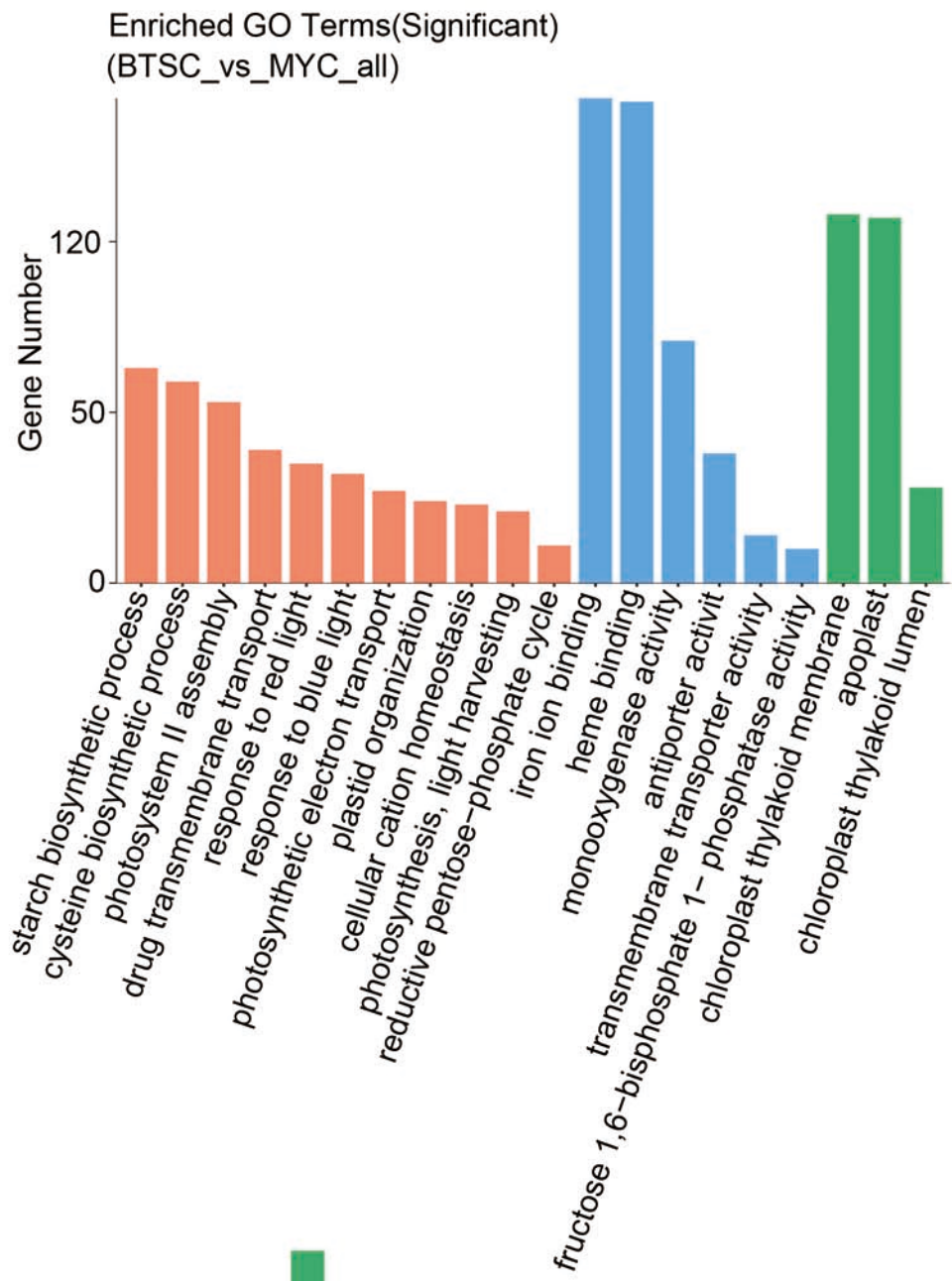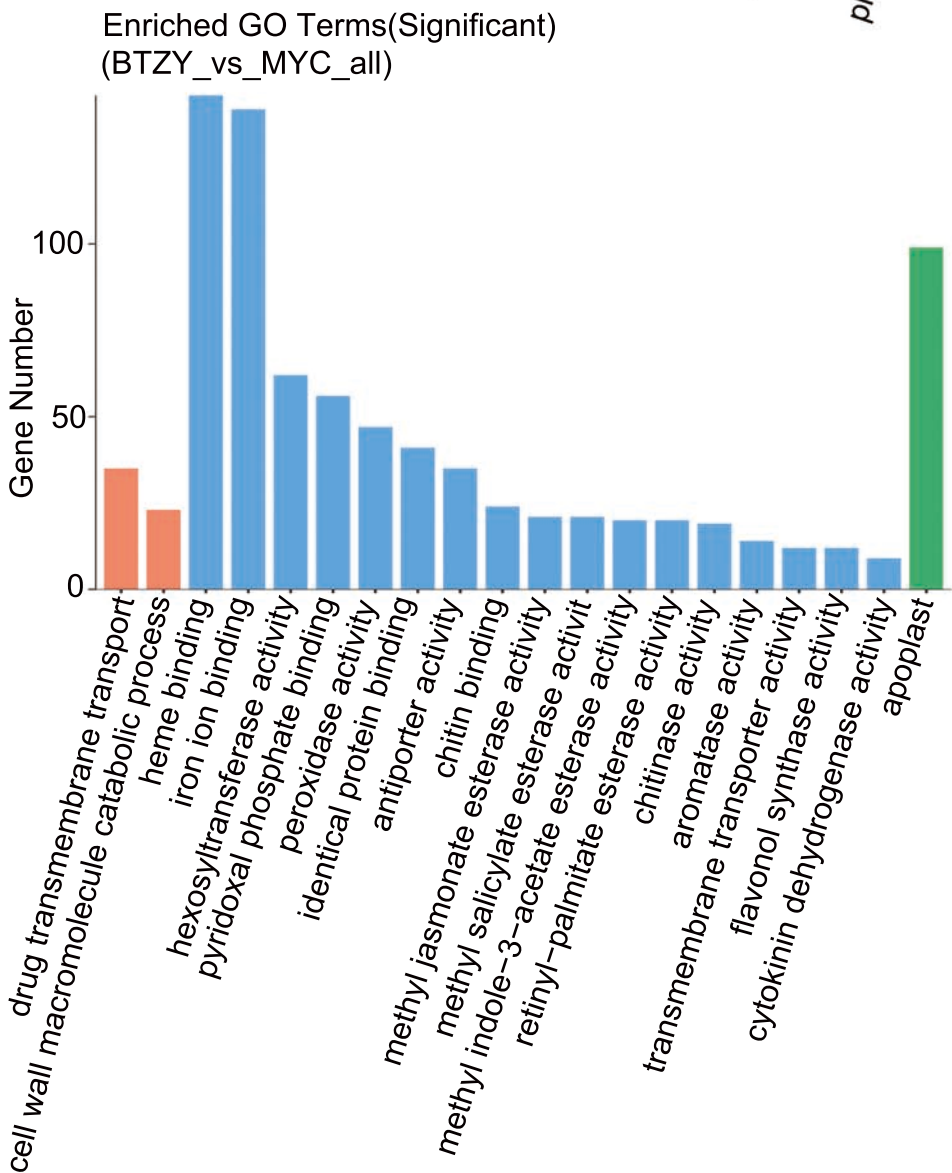

GO categories

- biological process (BP)
- molecular function (MF)
- cellular component (CC)

Supplement: Supplementary file 1 [file genes-13-01994-s001.zip › Figure S3.pdf]
